# The precautionary principle and dietary DNA metabarcoding: Commonly used abundance thresholds change ecological interpretation

**DOI:** 10.1111/mec.16352

**Published:** 2022-01-30

**Authors:** Bethan L. Littleford‐Colquhoun, Patrick T. Freeman, Violet I. Sackett, Camille V. Tulloss, Lauren M. McGarvey, Chris Geremia, Tyler R. Kartzinel

**Affiliations:** ^1^ Department of Ecology, Evolution, and Organismal Biology Brown University Providence Rhode Island USA; ^2^ Institute at Brown for Environment and Society Brown University Providence Rhode Island USA; ^3^ Yellowstone Center for Resources, Yellowstone National Park Mammoth Hot Springs Wyoming USA

**Keywords:** bighorn sheep, bison, frequency of occurrence, grazer‐browser continuum, herbivore, Hill numbers, microhistology, relative read abundance

## Abstract

Dietary DNA metabarcoding enables researchers to identify and characterize trophic interactions with a high degree of taxonomic precision. It is also sensitive to sources of bias and contamination in the field and laboratory. One of the earliest and most common strategies for dealing with such sensitivities has been to remove all low‐abundance sequences and conduct ecological analyses based on the presence or absence of food taxa. Although this step is now often perceived to be necessary, evidence of its sufficiency is lacking and more attention to the risk of introducing other errors is needed. Using computer simulations, we demonstrate that common strategies to remove low‐abundance sequences can erroneously eliminate true dietary sequences in ways that impact downstream inferences. Using real data from well‐studied wildlife populations in Yellowstone National Park, we further show how these strategies can markedly alter the composition of dietary profiles in ways that scale‐up to obscure ecological interpretations about dietary generalism, specialism, and composition. Although the practice of removing low‐abundance sequences may continue to be a useful strategy to address research questions that focus on a subset of relatively abundant foods, its continued widespread use risks generating misleading perceptions about the structure of trophic networks. Researchers working with dietary DNA metabarcoding data—or similar data such as environmental DNA, microbiomes, or pathobiomes—should be aware of drawbacks and consider alternative bioinformatic, experimental, and statistical solutions.

## 1 INTRODUCTION

Advances in dietary DNA metabarcoding have revolutionized our ability to address some of the most fundamental goals in ecology: to quantify the diversity of species and understand how they interact. Myriad technical developments in molecular ecology have improved our ability to identify foods, assess dietary diversity and overlap, and measure the relative abundance of taxa in the diets of wild animals, livestock, and humans (Deagle et al., 2009; Mata et al., 2019; Pegard et al., 2009; Reese et al., 2019; Valentini et al., 2009). Yet the utility of dietary DNA metabarcoding methods has occasionally come under scrutiny over doubts about the accuracy of a particular step in the bioinformatic pipelines that ultimately determine how we interpret the data.

Among the most controversial decisions about how to analyse dietary DNA has been how to convert sequence count data into dietary profiles. Early research primarily aimed to validate DNA metabarcoding as a method to generate accurate and precise lists of food species (Shehzad et al., 2012; Soininen et al., 2009; Valentini et al., 2009; Zeale et al., 2011). Researchers quickly became aware that contamination, PCR and sequencing errors, tag jumps, and biological and technical biases can complicate this aim (Pompanon et al., 2012). High‐throughput sequencing technologies generate thousands of low‐quality and erroneous sequences with each run. Since most errors occur at low relative abundances, truncating the long tail of the sequence abundance distribution clearly removes many such errors. Because eliminating errors is desirable, and because the goal of generating a list of food species can be accomplished using presence/absence data, an apparently simple solution was to eliminate low‐abundance sequences and conclude that the rest were “present” in a sample. Yet, despite the focus on presence/absence data, diet data sets often retained biologically meaningful signals of sequence relative read abundance (RRA) that could be corroborated by feeding trials (Deagle et al., 2009; Willerslev et al., 2014), stable‐isotopes analysis (Kartzinel et al., 2015), and microhistology (Soininen et al., 2009), albeit often with the need for correction factors (Thomas et al., 2016) or the lumping of sequences into operational taxonomic units (Clarke et al., 2014). Observations like these established awareness of the need to balance the aims of quantifying both the presence and relative read abundance of “true” food species against the risk of including errors.

Researchers continue to emphasize the occurrence of relatively abundant taxa because of the legacy of the early literature on presence/absence data, because it is an apparently simple solution to the risk of including errors, and because of the assumption that the most ecologically and nutritiously important foods are abundant. A common strategy is simply to remove taxa from a sample that do not exceed a minimum threshold. Dietary analyses may rely on minimum overall count thresholds (Valentini et al., 2009) or, more often in recent studies, sample‐wise RRA thresholds (Ait Baamrane et al., 2012; Kartzinel et al., 2015; Pompanon et al., 2012). Since removing sequences with low overall or sample‐wise RRA can eliminate low‐abundance errors, it is often characterized as a “conservative” option that minimizes the risk of including false‐positive sequences (Alberdi et al., 2018; Ando et al., 2020). Crucial drawbacks to this strategy, however, include the often arbitrary and subjective selection of thresholds, the inability to eliminate errors that exceed whatever threshold is selected, and the risk of inadvertently excluding true dietary taxa while inflating the apparent importance of the taxa that remain (Deagle et al., 2019; Kelly et al., 2019). There is thus much confusion about how to employ this strategy, and evidence of its efficacy is lacking, creating a need to focus on appropriate alternative strategies and the treatment of rare taxa (Ando et al., 2020).

What does it mean to be “conservative” with dietary DNA metabarcoding data? The answer to this question must be evaluated in the context of the goals of a particular study. More than a decade ago when the predominant goal in this field was to evaluate whether a particular taxon was present in a sample, it may have been prudent to discard low‐abundance sequences as putative contaminants in order to avoid including spurious taxa. On the other hand, there is a risk in removing low‐abundance sequences that could represent rare food taxa and provide information about animal nutrition, foraging behavior, and the structure of food webs. Understanding the ecology and evolution of dietary specialization, for example, requires differentiation of dietary specialists that concentrate on one or a few resources from dietary generalists that utilize a more even array of resources (Araújo et al., 2011; Bolnick et al., 2003). Understanding how individual feeding interactions scale‐up to establish the links and nodes of complex trophic networks requires determination of interaction strengths, and whether “weak” links are as important to the network as they are often theorized to be (Pringle & Hutchinson, 2020). Real sources of variation in dietary breadth and interaction strength will necessarily translate into variation in the number of rare dietary DNA sequences that appear in data, and this in turn ensures that any bioinformatic decision about how to treat low‐abundance sequences can differentially impact the dietary profiles of animals with broad versus narrow diets. Awareness of how common strategies for analyzing low‐abundance sequences distort dietary profiles and alter ecological interpretations is critical for the appropriate treatment of data.

## 2 DIFFERENTIAL IMPACTS OF DATA FILTERING ON SIMULATED DIETS

Although abundance‐filtering is common in DNA metabarcoding pipelines, the RRA cutoff used to determine which taxa count as “present” varies widely among studies (Alberdi et al., 2018; Pompanon et al., 2012). Deagle et al. (2019) suggested that a 1% RRA threshold may be suitable for many dietary studies, but values in the range of 0%–5% are not uncommon (Alberdi et al., 2018; Bohmann et al., 2018; Kartzinel et al., 2019; Komura et al., 2018; ter Schure et al., 2021).

### 2.1 Dietary profile simulations

**FIGURE 1:**
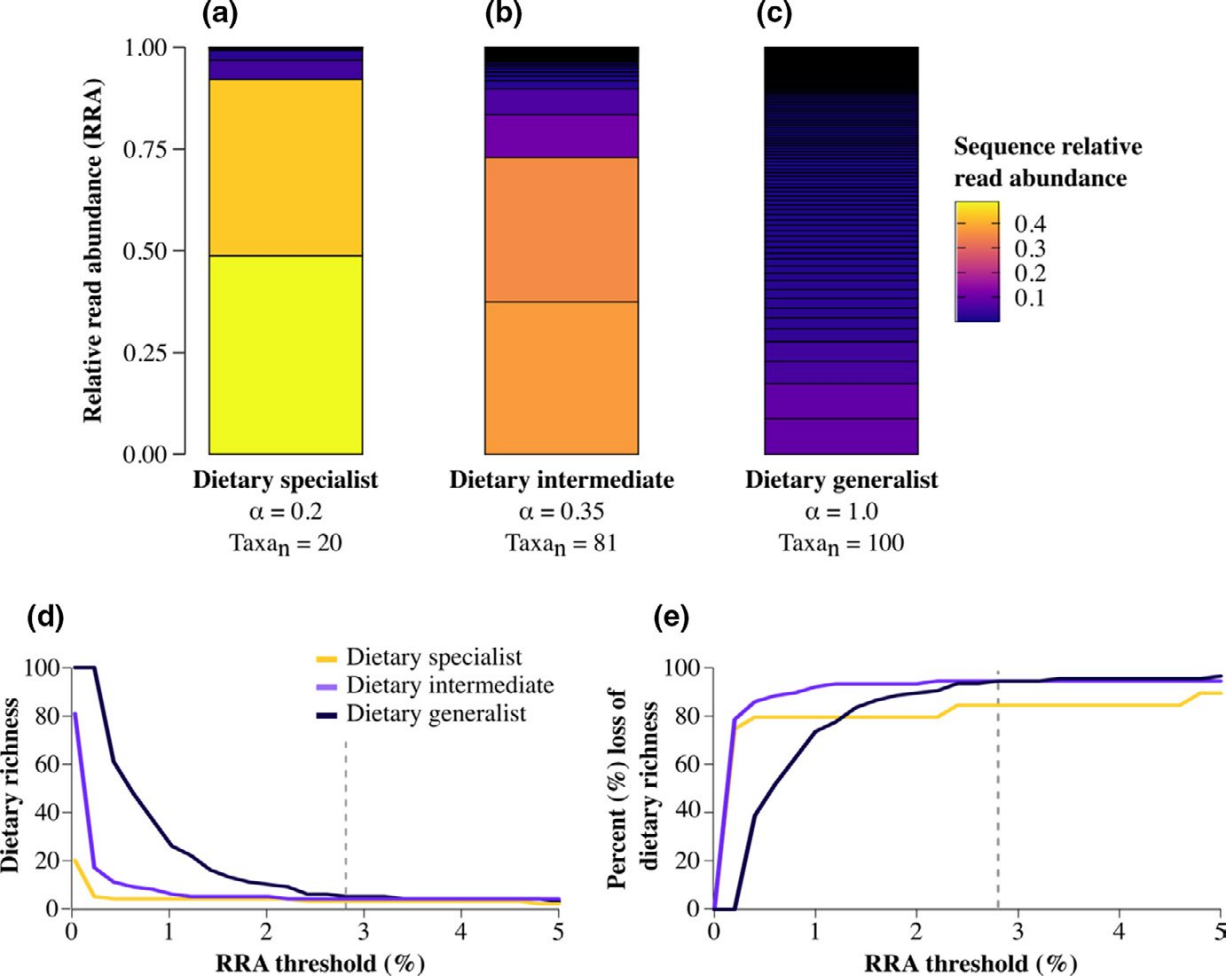
Different impacts of RRA thresholds on simulated specialist and generalist diets. The dietary profiles of a (a) specialist, (b) intermediate, and (c) generalist feeder as simulated using Pareto distributions. When the shape parameter (α) and total number of food taxa (Taxa_n_) are low, there is a large skew in the rank‐abundance distribution (i.e., few food taxa with high relative abundance); increasing these values increases the richness and evenness of the dietary profile (i.e., many food taxa, each with lower relative abundance). In each stacked barplot, the color of each segment represents the relative abundance of each simulated taxon in the diet profile. Increasing the threshold from 0% to 5% for each diet profile resulted in differential impacts on the (d) inferred dietary richness and (e) % loss of initial dietary richness from each sample. A 2.8% threshold (grey dashed lines in d and e) results in similar levels of inferred dietary richness and % losses of taxa across samples

Consider variation in both the richness and rank‐abundance distribution of animal diets. Diets are generally characterized by skewed distributions that are concentrated on a small number of predominant resources but also include many rare resources (Forister et al., 2015). These heavy‐tailed distributions can be approximated by a power law function known as the Pareto distribution (Appendix S1 provides detailed simulation methods). Dietary specialists, such as the koala (*Phascolarctos cinereus*), usually consume a very small number of food taxa, and this type of dietary profile can be represented by a highly‐skewed distribution (e.g., Figure 1a). In contrast, dietary generalists, such as the racoon (*Procyon lotor*), tend to consume a wide variety of resources that can be represented by a more even distribution (e.g., Figure 1c). The skewness of a Pareto distribution is denoted by a shape parameter, *α* (Figure S1).

To develop theoretical intuition, we simulated diet profiles of generalist and specialist consumers (Appendix S1). In our simulations, we considered a set of three hypothetical consumers with access to an identical food base comprising 100 potentially suitable taxa. We assumed that each consumer differed only in the specificity with which it consumed food taxa. This reflects the aims of many empirical studies to characterize feeding specializations, food preferences, foraging behaviors, competition, or other ecological factors that influence the composition of diets and the structure of trophic networks (Figure 1a–c; Appendix S1). For each consumer, the probability that each of the 100 food taxa would be selected was calculated using the R package Pareto v.2.3.0 (Riegel, 2018). To generate diet profiles that differed in their degree of specialism, we defined probability distributions using skew values to represent specialist (*α* = 0.20), intermediate (*α* = 0.35), and generalist (*α* = 1.00) diets (Appendix S1, Figure S2). From each of these probability distributions, we made 25,000 draws to simulate a common number of DNA metabarcoding sequence‐reads obtained per sample with an Illumina MiSeq. The resulting diets are thus theoretically free of any differences in sampling, contamination, technical bias, and error—they represent an ideal data set in which draws of taxa are made in proportion to their true abundances. The specialist's diet profile comprised a much narrower subset of the 100 available resources compared to the intermediate and generalist feeder (Figure 1a–c).

### 2.2 Skewed diet profiles are differentially impacted by the same thresholds

To evaluate the theoretical impact of removing low‐abundance taxa from the three simulated diet profiles, we incrementally filtered taxa that did not exceed thresholds of 0%–5% RRA (Appendix S1). We found that abundance‐filtering differentially impacted the relatively skewed diet profiles of specialist and intermediate feeders compared to the relatively even diet profile of the generalist (Figure 1). Incrementally removing taxa resulted in a large decrease in richness for each sample, but an immediate 75%–80% loss of dietary richness occurred after applying only a mild 0.2% filter to the specialist and intermediate diet profiles, respectively (Figure 1d–e). In contrast, the generalist diet profile did not exhibit any reduction in richness until more stringent thresholds were applied (Figure 1d–e). Then, at an intermediate threshold of 2.8%, diet profiles converged to generate the incorrect impression that all samples had similar richness (Figure 1d; grey dashed line). At this level, there was a >95% reduction in the inferred richness of the intermediate and generalist diets (Figure 1e; grey dashed line). All else equal, these simulations reveal how any threshold can have a qualitative impact on the apparent richness and composition of a sample in ways that differ depending on its true composition.

## 3 DIFFERENTIAL IMPACTS OF DATA FILTERING ON WELL‐STUDIED WILDLIFE DIETS

We sought to illustrate how abundance‐filtering can qualitatively alter interpretations of real dietary DNA metabarcoding data by testing predictions about (*i*) seasonality and (*ii*) trait‐based differences in the diets of large herbivores from Yellowstone National Park (Appendix S2 provides detailed methods for wildlife diet analysis). Following a well‐established annual migration, Yellowstone bison (*Bison bison*) and bighorn sheep (*Ovis canadensis*) follow springtime plant green‐up from their lower elevation winter range to higher elevation meadows. We predicted both species would exhibit greater dietary richness in summer compared to winter because: both species graze on graminoids year round; both have access to a larger number of plant species in summer; both incorporate species‐rich suites of forbs into summer diets; and both incorporate species‐poor shrubs into winter diets (Bergmann et al., 2015; Craine, 2021; Geist, 1971; Peden et al., 1974; Wagner & Peek, 2006). We further predicted that bison would have greater dietary richness than bighorn sheep because although both species have ruminant digestive systems, bison are much larger (~625 vs. 75 kg body mass), have wider muzzles, and have larger home ranges. Thus, all else equal, bison should exhibit greater dietary richness because each individual will encounter and/or be able to consume a greater variety of available forage (Clauss et al., 2013). Yet despite firmly established allometric differences in digestive physiology that support our hypothesis, recent studies of African ungulates have reported a lack of correlation between dietary richness and body size (Kartzinel et al., 2015; Kartzinel & Pringle, 2020). The taxonomic precision of DNA metabarcoding could help reconcile such cases where established allometric and foraging ecology theories appear to diverge from data, and our results will show how crucial it is to consider the potential effects of abundance‐filtering on these types of downstream ecological interpretations.

Our illustrative analyses of bison and bighorn sheep diets are based on 35 samples from winter and summer (median = 10 per species per season) analyzed by sequencing the P6 loop of the chloroplast *trn*L(UAA) intron and using publicly available plant reference data (Appendix S2). We required a 100% match between a dietary sequence and a reference sequence that was present in the library to include a plant taxon in our analysis, thereby minimizing the risk of introducing sequencing errors and chimeras. This mapping strategy is reasonable when researchers have access to an extensive reference library of potential food sequences—enabling accurate sequence identification and efficient error elimination—although it may not be as appropriate for studies involving markers and/or taxa with poor reference coverage compared to what is available for *trn*L‐P6 (Pompanon et al., 2012). Overall, 91.1% of high‐quality sequence reads were mapped to the reference library across all 35 samples (1,071,130 of 1,175,453 sequences overall reads; median = 93.2% per sample). From these mapped reads, we initially characterized 357 plant sequences and retained a subset of 355 sequences after rarefying samples to equal sequencing depth (Dryad https://doi.org/10.5061/dryad.kwh70rz4s; Data S1), with 88% identified to family (312 of 355 sequences), 55% to genus (196 of 355), and 23% to species (82 of 355). Because the resulting diet profiles comprise only those taxa that match a reference plant sequence, they could represent an underestimate of the true plant richness in a sample given that: (*i*) a subset of available plant species may not yet be included in public data and/or (*ii*) the *trn*L‐P6 marker has a limited ability to differentiate among certain closely related species (Taberlet et al., 2007). Importantly, if these diet profiles underestimate the true dietary richness, it will be due to limitations inherent to the marker and available reference data rather than an artefact arising from data‐filtering decisions.

Following the same procedure that we applied to simulated diets, we filtered taxa from samples using 0%–5% RRA thresholds (Appendix S2). We compared differences in the inferred dietary richness within and between species based on individual samples, based on the average richness across samples, and based on the total richness of each population after accounting for differences in sample size. We observed that thresholds can: (*i*) alter the rank‐order of inferred richness values, (*ii*) obscure patterns of seasonal variation within and among species, and (*iii*) obscure differences in the overall dietary breadth of species.

### 3.1 Thresholds alter rank‐order of dietary richness

**FIGURE 2:**
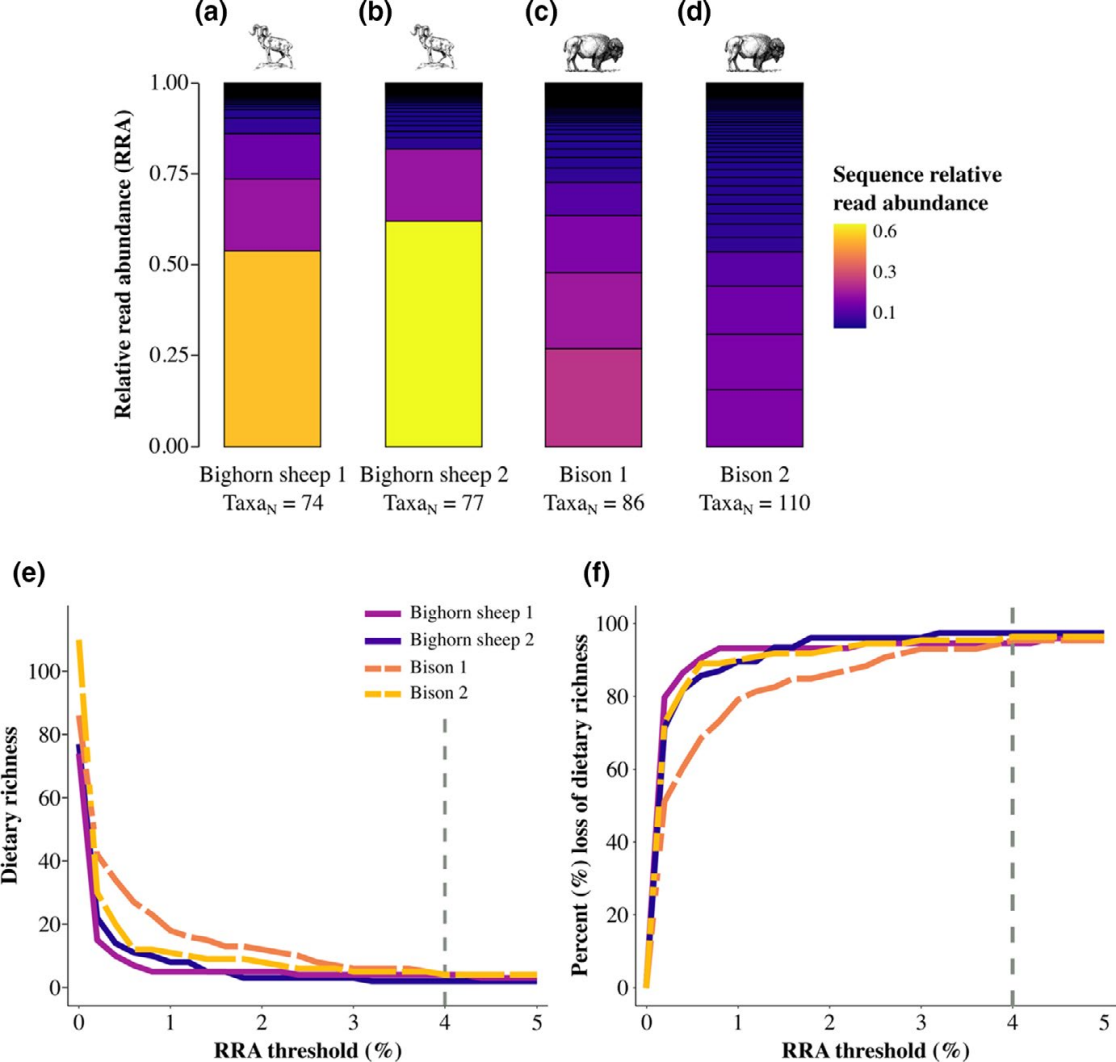
Using RRA thresholds differentially impacted samples that varied in evenness, altering the inferred rank‐order of dietary richness. Stacked barplots show four representative dietary DNA metabarcoding profiles from (a, b) bighorn sheep and (c, d) bison in summer. The color of each segment represents the relative abundance of a taxon prior to abundance‐based filtering. Increasing the minimal RRA threshold from 0% to 5% for each dietary profile resulted in different impacts on (e) the inferred level and rank‐order of dietary richness across samples as well as (f) the % loss of dietary richness. A 4% threshold (grey dashed lines in e and f) resulted in similar levels of inferred richness and % losses of taxa across samples

Detailed comparisons of four representative samples show both intra‐ and interspecific variation in the shape of diet profiles (Figure 2a–d). Differences in richness and evenness led to changes in the inferred rank‐order of samples as low‐abundance taxa were removed (Figure 2e), just as they did in the simulation study. The dietary richness of each sample decreased by ≥51% with a mild threshold of 0.2%. The sample with the lowest initial richness exhibited the largest apparent decline in richness (80% loss; Figure 2f). A larger threshold of 4% resulted in the conversion of both dietary richness and the percent loss of initial richness across all samples, regardless of the consumer species (grey dashed lines in Figure 2e–f). Thus, applying bioinformatic thresholds to real data reproduced the patterns observed in theoretical simulations and obscured ecologically meaningful differences in the shape of diet profiles: (*i*) a mild threshold led to a precipitous drop in richness for diet profiles with relatively narrow breadth, (*ii*) a more stringent threshold led to convergence in apparent richness between broad and narrow profiles, and (*iii*) different thresholds altered the rank‐order of dietary richness that was inferred for this set of consumers.

### 3.2 Thresholds obscure seasonal patterns dietary richness

**FIGURE 3:**
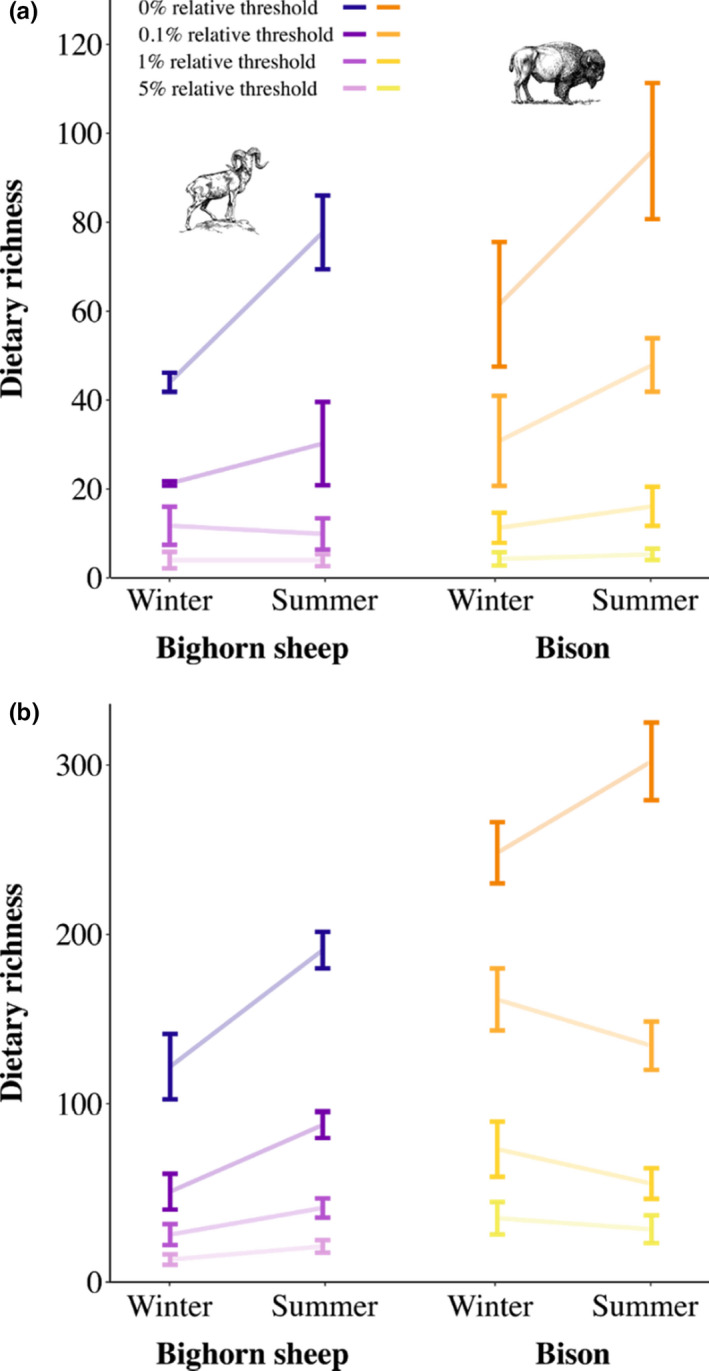
Thresholds altered ecological patterns in dietary DNA metabarcoding data. We compared (a) mean dietary richness and (b) total population‐level dietary richness of bighorn sheep and bison in summer and winter. Total population‐level dietary richness was estimated for winter and summer based on extrapolation to double the minimum seasonal sample size for each species (*N* = 8 bighorn sheep; *N* = 20 bison). Error bars represent (a) standard deviations and (b) 95% upper and lower confidence intervals. In all plots, lines connect mean dietary richness for winter and summer at each relative threshold

Incrementally removing low‐abundance taxa eroded the apparent increase in mean dietary richness across samples from winter to summer. The mean richness of bighorn sheep and bison diets was greater based on the totality of the sequence data in summer versus winter, but this seasonal difference disappeared when low‐abundance taxa were removed and only the subset of dominant dietary taxa remained (Figure 3a). With each threshold, there was a “dropout” of low‐abundance grasses (family Poaceae), buckwheats (Polygonaceae), evening primroses (Onagraceae), and roses (Rosaceae) in the diets of both species, in both summer and winter (Data [Link], [Link]). In summer, however, there was a disproportionate loss of taxonomically diverse sequences: bighorn sheep samples lost sequences representing mustards (Brassicaceae) and legumes (Fabaceae; Data S2), while bison lost many sedges (Cyperaceae) and willows (Salicaceae; Data S3). Sequences remaining following the most stringent thresholds included a subset of common foods for both bighorn sheep (e.g., grasses, geraniums (Geraniaceae), and roses; Data S2) and bison (e.g., sedges and grasses; Data S3). Thus, although using thresholds in this way may help researchers identify the “core” resources present, it can also generate the false impression that consumers have narrow diets, disproportionately exclude taxa from species‐rich groups (e.g., grasses, forbs), and generate artificially simple trophic networks.

### 3.3 Thresholds obscure population‐level differences in overall dietary breadth

The removal of low‐abundance taxa differentially influenced each species’ total dietary richness. As above, incremental removal of taxa resulted in a large decrease in total richness inferred for both bighorn sheep and bison (Figure 3b, Data [Link], [Link]). Dietary richness apparently increased from winter to summer for bighorn sheep, regardless of threshold. In contrast, seasonal differences in dietary richness were apparently reversed for bison, depending on threshold: total richness appeared greater in summer when no threshold was applied, but it appeared greater in winter whenever low‐abundance taxa were removed (Figure 3b). This drop in summer dietary richness for bison was driven by the removal of a large number of taxa, each occurring at low relative abundance: asters, evening primroses, broomrapes (Orobanchaceae), and buttercups (Ranunculaceae; Data S5). A purported benefit of population‐level analyses is the ability to “average out” sampling stochasticity, since a resource that is erroneously excluded from one sample may still be registered in another for downstream analyses (Deagle et al., 2019; Kartzinel & Pringle, 2020). Our results illustrate the risk of presuming this outcome when consumers eat many food taxa that each represent a relatively small proportion of the diet.

### 3.4 Ecological interpretations

Apparent declines in richness with each threshold had the potential to alter support for our hypotheses. We predicted dietary richness would be maximized in summer for both species and found strong support for this prediction in the complete data set (Figure 3). Abundance‐filtering, however, eroded this seasonal pattern and, in the case of bison, reversed it (Figure 3). Similarly, we predicted bison would have greater dietary richness than bighorn sheep; we observed this difference with the complete data set, but abundance‐filtering eroded or eliminated it (Figure 3).

Evaluating bioinformatic strategies in light of natural history can guide interpretation. Ideally, information from comprehensive DNA barcode libraries would support taxonomic inference and facilitate error‐filtering (Pompanon et al., 2012). Although we do not yet have a comprehensive library for the flora of Yellowstone, every sequence in our complete data set was a 100% match to publicly available sequence data and the overall diet composition was realistic for the animals we studied. Tracking taxa that dropped out of the data set with each threshold enabled us to evaluate patterns (Data [Link], [Link], [Link], [Link]). For example, wind‐dispersed pollen deposition is thought to be a common source of low‐abundance sequence contamination (Ando et al., 2018). However, we observed dropout of some wind‐dispersed taxa that are palatable to these herbivores using a mild threshold (e.g., some grasses, sedges) while other wind‐dispersed taxa from less palatable groups exceeded high thresholds (e.g., pine trees, Pinaceae). We observed this in summer when plants reproduce, and pollen contamination is likely, as well as in winter when plants are reproductively dormant. Although we cannot say definitively for any given sample whether a sequence represents a food that was deliberately eaten by the animal, we can use the observed variation in sequence representation to guide ecological interpretations. Indeed, quantifying this variation is a fundamental goal of dietary DNA metabarcoding research.

## 4 STRATEGIES FOR IMPROVEMENT

There is nothing inherently wrong with excluding low‐abundance taxa under at least two conditions: (*i*) the objective is to identify a subset of “core” taxa that occur above a threshold or (*ii*) the rare sequences represent errors that persist in the data despite experimental controls and other bioinformatic strategies. Although DNA metabarcoding is useful for identifying core dietary taxa (scenario *i*), most studies aim to elucidate more complete diets and trophic networks (Ando et al., 2020). There is thus a need to ensure that any suite of rare sequences to be analyzed are as free of error as possible, although this can be difficult to verify (scenario *ii*). Because there have been many improvements to sampling strategies and laboratory protocols that reduce the incidence of contamination (Alberdi et al., 2018; Ando et al., 2020; Creer et al., 2016; Mata et al., 2019; McInnes et al., 2017), we will focus on promising and transparent analytical strategies to account for low‐abundance taxa.

### 4.1 Computational and mathematical alternatives to arbitrary thresholds

Promising strategies provide alternatives to relying on arbitrary abundance thresholds. For example, the flexible simulation strategy we demonstrated above may be generally useful for evaluating the effects of analytical options on downstream ecological interpretations. This could include power analyses to assess the probability of detecting an effect or sensitivity analyses that evaluate any qualitative changes to the conclusion of a study that would result from different filtering strategies (Kartzinel et al., 2015; Kartzinel & Pringle, 2020). Related algorithms developed specifically for dietary DNA metabarcoding have potential to address drawbacks of arbitrary thresholds directly. For example, Bayesian strategies can be used to assess underlying uncertainties in the identification of “true” sequences in a food web rather than assuming the suitability of a fixed cutoff (Cirtwill & Hambäck, 2021).

Even without using RRA filters to purge rare sequences from analysis, it is possible to manage their impact on interpretations. For example, Hill numbers are a mathematically unified family of diversity indices that quantify diversity in units of effective number of species (Hill, 1973), enabling researchers to upweight or downweight rare taxa using the scaling parameter *q* (Jost, 2006). The larger the *q* value, the greater the importance attributed to abundant taxa. The lowest *q* value is 0, which is equivalent to species richness (all taxa are counted equally). Increasing to *q* = 1 yields a value equivalent to the exponential of Shannon entropy (taxa are weighted in proportion to abundance without disproportionately favoring either rare or abundant ones). Finally, *q* = 2 equals the inverse of Simpson concentration, which upweights abundant taxa and discounts rare ones (Chao et al., 2014). Hill exponents can, therefore, be considered as the richness of all species (*q* ^0^), the diversity of "typical” species (*q* ^1^), or the diversity of dominant species (*q* ^2^). When rare taxa are considered to be of low importance, regardless of whether they are thought to be real or errors, researchers can use higher *q* values to downweight them (Alberdi & Gilbert, 2019). In contrast, if rare taxa are considered essential for proper understanding, researchers may need to rely on exact counts of taxa present (Alberdi & Gilbert, 2019).

**FIGURE 4:**
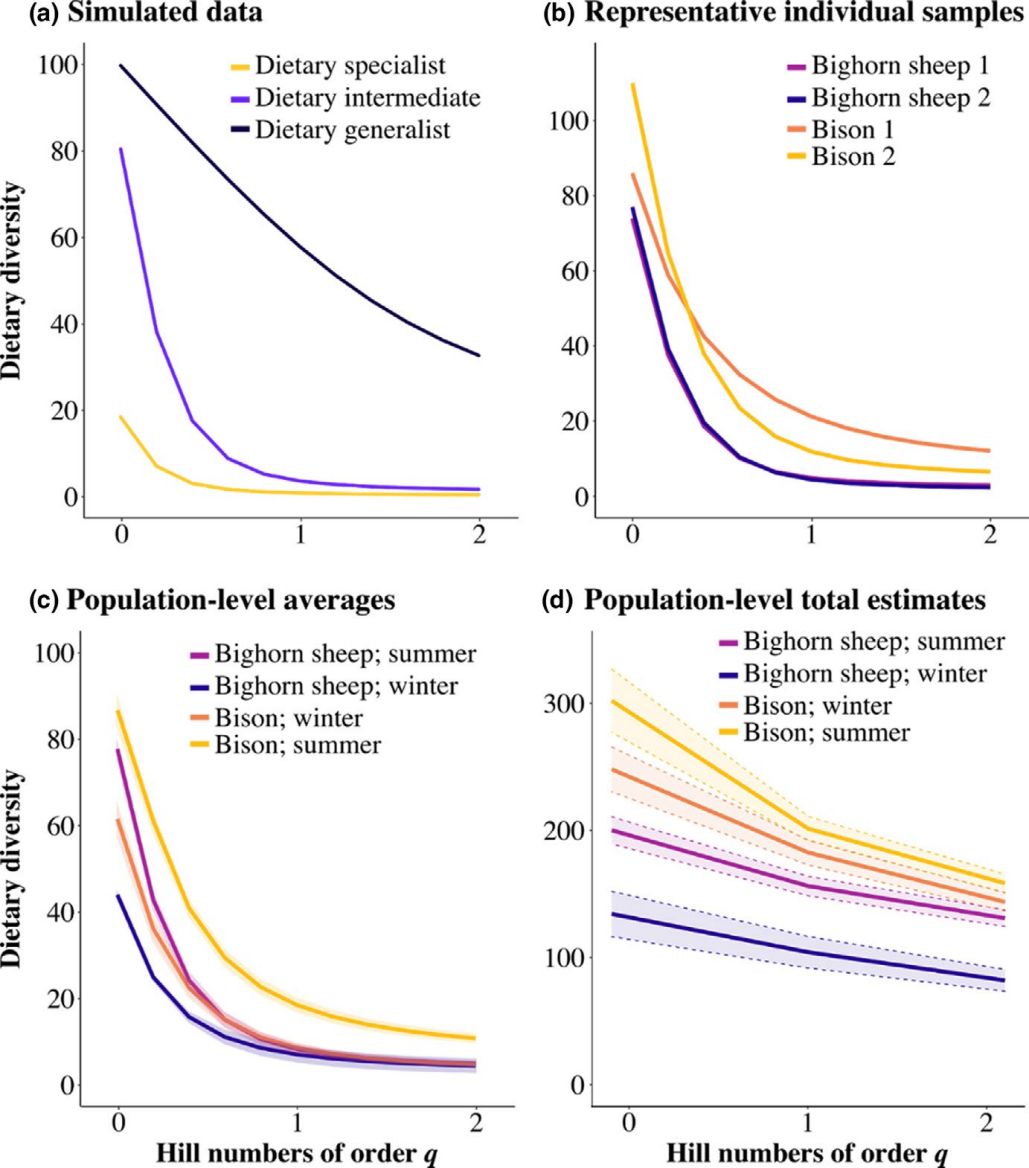
Hill numbers applied to both simulated and real dietary DNA metabarcoding data. All curves show a decline in apparent dietary diversity with increasing *q* due to the increasing emphasis on abundant taxa. Curves show how sensitive each set of diet profiles is to increasing *q* based on (a) simulated dietary profiles using different Pareto distributions (Figure 1), (b) a set of representative samples from Yellowstone (Figure 2), (c) the average population‐level values from Yellowstone (Figure 3a), and (d) the total population‐level estimated values from Yellowstone (Figure 3b). In contrast to the effect of applying RRA thresholds to the same data, these curves convey more information about the relative abundance of both common and rare taxa while retaining clearer rank‐order of samples

To illustrate similarities and differences in using Hill numbers to downweight rare taxa compared to using thresholds to exclude them, we applied this method to our simulations and our Yellowstone data (Appendix S2). When downweighting emphasis on rare taxa from *q* ^0^ to *q* ^2^, the apparent diversity of food taxa converged asymptotically towards a low value (Figure 4). This familiar pattern is superficially similar to applying incremental thresholds to the data. In contrast to thresholds, however, Hill numbers had the desirable quality of retaining the rank‐order of samples. This contrast is especially evident in comparisons of simulated diet profiles with known dietary breadth, since the rank‐order of the theoretical generalists and specialists was retained across all values of *q* but was inverted by the application of RRA thresholds (Figures 1e and 4a).

### 4.2 Multiple datastreams can corroborate and contextualize

Comparing independent datastreams can support inferences in dietary studies (Nielsen et al., 2018). Ideally, researchers would qualitatively corroborate conclusions in light of strengths and weaknesses inherent to each method. Prior studies, for example, have combined DNA metabarcoding with direct feeding observations or experiments (Deagle et al., 2010; Thomas et al., 2014; Willerslev et al., 2014), stable isotope analysis (Craine et al., 2015; Kartzinel et al., 2015), and gut content analysis or microhistology (King & Schoenecker, 2019; Newmaster et al., 2013; Soininen et al., 2009).

To help contextualize our results for bison and bighorn sheep, we compared dietary diversity indices obtained from DNA metabarcoding with corresponding microhistology data. Microhistology is a method involving the visual examination of fecal material on slides (Appendix S2). An advantage of microhistology is the opportunity to identify taxa that may not be covered by the *trn*L‐P6 marker we used in this study, such as lichens, mosses, and fungi. However, microhistology is so labour intensive that it is common to: (*i*) only identify taxa to higher taxonomic classifications (e.g., our analysis aimed to identify plant fragments to genus); (*ii*) group species into broad taxonomic or functional groups (Garnick et al., 2018; Mayes & Dove, 2000); (*iii*) underestimate the proportion of digestible forbs (King & Schoenecker, 2019; Shrestha & Wegge, 2006); and (*iv*) lump fecal deposits into “representative” samples rather than analyzing each individually.

**FIGURE 5:**
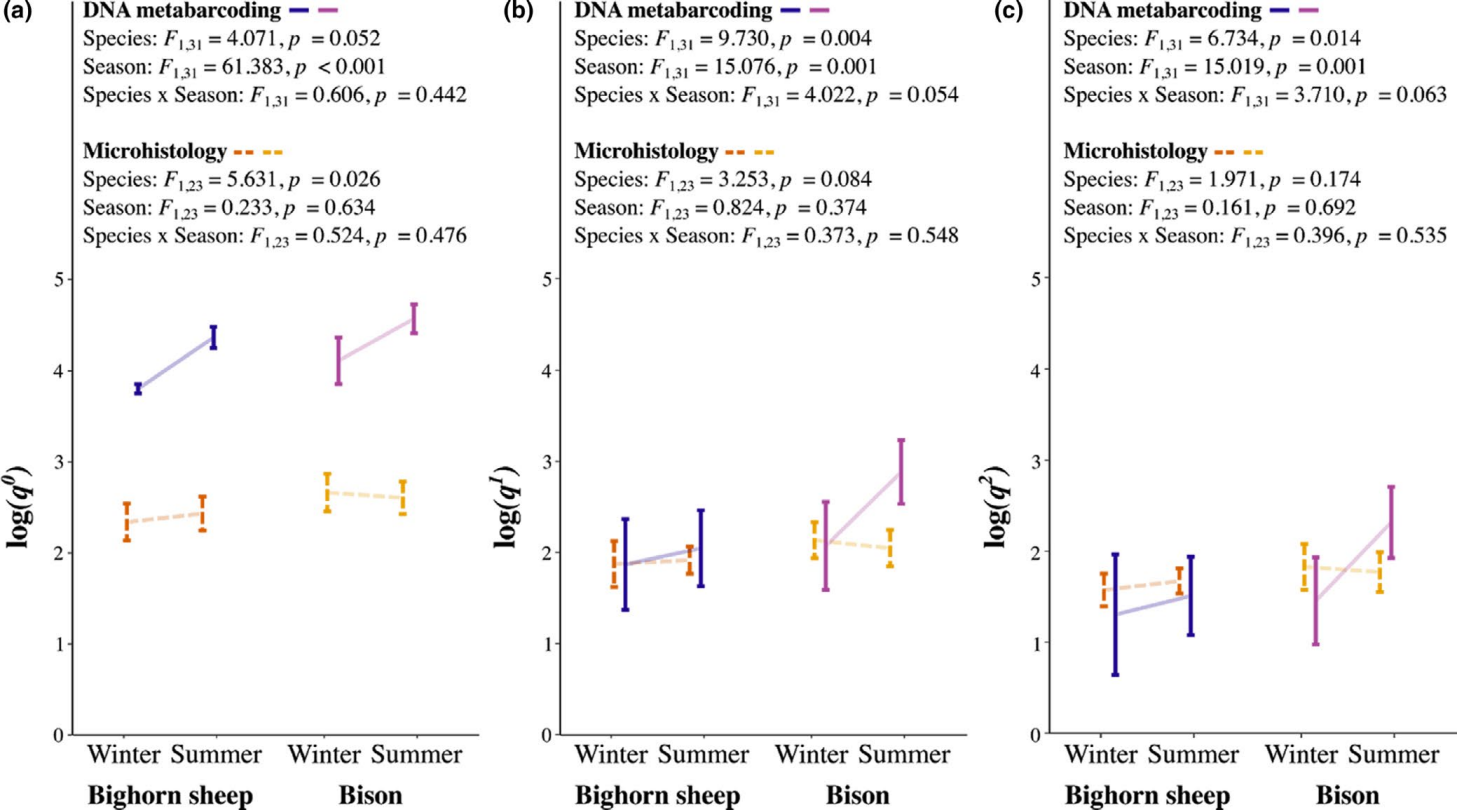
Seasonal changes in dietary diversity based on DNA metabarcoding and microhistology. For both bighorn sheep and bison, we compare log‐transformed (a) dietary richness (*q* ^0^), (b) the number of “typical” (*q* ^1^), and (c) the number of dominant (*q* ^2^) plant taxa identified in DNA metabarcoding data (dark solid lines) and microhistology data (light dashed lines). Lines connect the mean values with error bars that represent standard deviations. For microhistological analysis, when multiple samples were collected per herd per season, these samples were pooled into a composite scan of fecal material

Dietary DNA metabarcoding was better able to differentiate closely related food taxa than microhistology and thus revealed a greater diversity of foods. Consider the 58 grass genera known to occur in Yellowstone (Whipple, 2001). Microhistology identified only nine grass taxa with genus‐level precision across all samples and seasons (Data S6, Figure S4), but DNA metabarcoding revealed between 22 and 40 grass DNA sequences (representing from 5 to 9 grass genera) across each species, depending on season (Data [Link], [Link]). In principle, this means that up to 49 grass genera could be lumped into the “unknown Poaceae” category of our microhistology data. While microhistology provides a reasonable estimate of overall “grass” contributions to diets, it masks the contribution of each constituent grass species to diversity. Microhistology suggested that bison had greater year‐round dietary richness (*q^0^*) compared to bighorn sheep, although there was no significant difference in the number of typical (*q* ^1^) or dominant (*q* ^2^) plant taxa (Figure 5). In contrast, DNA further illuminated a significant effect of species and season on the typical (*q* ^1^) and dominant (*q* ^2^) number of plant taxa consumed by bison and bighorn sheep (Figure 5). Importantly, results obtained from both methods aligned closely when we used Hill numbers to downweight rare taxa (Figure 5). Taken together, these results support the interpretation that many rare taxa were present in the dietary DNA and accounting for them provided a finer level of detail that would not otherwise be possible.

### 4.3 Transparency and reproducibility

Awareness of how data manipulations influence downstream analyses is vital to ensuring transparency and reproducibility. Given that different decisions about whether and how to use RRA‐based filters in bioinformatic pipelines has the potential to alter ecological patterns, transparency will be crucial for high‐resolution dietary data to accumulate in ways that make it a useful scientific resource. To ensure transparency, it is helpful to differentiate between the bioinformatic steps that are initially used to filter a data set of errors from any subsequent summaries of the data or statistical analyses. In the literature, RRA‐based filters have been used both for both purposes—to purportedly eliminate low‐abundance contaminants and to summarize the relatively abundant “core” resources—which complicates comparisons. Whereas many journal polices stipulate that DNA metabarcoding studies should publish tables of sequence read data, a review by Ando et al. (2020) revealed that 46% of studies omitted them. Providing a quality‐ and contaminant‐filtered count table of sequence data that has not otherwise been transformed, rarefied, or filtered to remove low‐abundance sequences can help reviewers and other researchers evaluate interpretations that may be sensitive to the presence of rare sequences. Such tables are needed to enable further analyses, including those based on statistical frameworks designed specifically for sequence count data (e.g., DESeq2; Love et al., 2014). However, the literature may often give the impression that such data sets are filtered incompletely and potentially wrong. Complicating matters, researchers are increasingly able to work with commercial laboratories to generate dietary DNA profiles and need to be aware that these services often apply low‐abundance filters by default. Good communication between purveyors and practitioners is critical, especially when detection of minor dietary components is needed (Scasta et al., 2019). Providing access to more complete data sets does not preclude researchers from generating, analyzing, and sharing filtered versions of the data—but it does offer the advantage of transparency.

## 5 DISCUSSION

The precautionary principle emphasizes caution and review before acting in the absence of conclusive evidence to support a decision (Van Der Sluijs et al., 2005). In the context of DNA metabarcoding, researchers face a dilemma: all else equal, will it tend to be better to include or exclude rare DNA sequences from analysis? Researchers initially suggested that prudence dictates a need to gather more evidence before including low‐abundance sequences in analysis—excluding such sequences has thus often been framed as conservative (Brown et al., 2015; Pompanon et al., 2012). We argue that it may often be prudent to retain rare sequences and weigh them appropriately based on relative abundance rather than risk obscuring real patterns in the data—that the rarity of a sequence alone is insufficient evidence of an error or otherwise unimportant trophic link to justify its removal. Advances in conceptual and methodological approaches for DNA metabarcoding now offer alternatives to simple abundance‐filtering that could help researchers better balance these risks and support more robust ecological interpretations.

Prior empirical evaluations of abundance‐filtering in dietary and environmental DNA studies have shown that many contaminants (but not all) tend to occur at low relative abundance (Alberdi et al., 2018; Ando et al., 2018). By quantifying plant DNA contamination in samples from herbivores fed controlled diets, for example, Ando et al. (2018) noted that removing sequences below 1% RRA reduced the probability of incorporating contaminants. There are at least two ways to reconcile experimental results like this with the issues we illustrated here: (*i*) researchers may benefit from obtaining empirical estimates of contamination rates and sources that they can use as evidence to support decisions about what sequences to exclude; (*ii*) since contaminants rarely rise to high relative abundance, they represent a source of error that can be managed in many research applications by using appropriate statistics and by weighing sequences based on RRA. Importantly, although a certain threshold may be shown to reduce the probability of including contaminants in one study, it does not necessarily follow that it is a procedure that eliminates all contaminants or retains all relevant sequences. Similar thresholds could be inappropriate in other studies since contamination rates vary among target taxa, environments, barcode regions, and methods (Ando et al., 2020). Hence, focus on how to appropriately account for rare sequences is warranted.

We began this paper by asking when and whether using thresholds to remove low‐abundance sequences makes sense, noting the importance of addressing this question with respect to a particular research objective. If the aim is to determine whether the DNA of a particular taxon is definitively present in a sample, then it might be appropriate to remove low‐abundance sequences. If, however, the goal is to compare dietary profiles or infer the topology of trophic networks, then it may be risky to abundance‐filter data in light of the evidence that removing low‐abundance taxa can distort comparisons of interest. In particular, removing rare taxa from the diet profiles of species with a relatively high degree of individual diet specialization—including predators and phytophagous insects (Bolnick et al., 2007; Codron et al., 2016)—is liable to artificially accentuate the inferred level of interindividual variation by omitting overlapping food sources from a subset of diet profiles. In contrast, the effect on diet profiles from populations with less inter‐individual variation—such as large mammalian herbivores that tend to have broader and more even diet profiles (Codron et al., 2016)—could be to eliminate sources of variation in ways that artificially homogenize the group.

Accounting for rare food taxa can be integral to understanding animal diets. Wild species are often observed consuming unexpected food items, which can inspire fascination and challenge long‐standing dogma (Burton, 2018). For example, many species are unable to obtain vital trace elements through “normal” diets (Pringle & Hutchinson, 2020). Crocodilians are generally assumed to be obligate carnivores incapable of digesting plant proteins and polysaccharides, but yet they regularly consume fruits to supplement an otherwise carnivorous diet (Platt et al., 2013). Many ungulates, in contrast, are thought to be obligate herbivores but yet surprising examples of protein‐rich food sources are regularly documented, including deer eating songbirds (Pietz & Granfors, 2000) and warthogs hunting antelope (Roberts, 2012). Large mammalian herbivores are able to feed on a broad array of food plants, but nevertheless feed preferentially on a subset of available plant species while avoiding others (Owen‐Smith & Novellie, 1982). Animals may rarely eat desirable foods if they benefit from diversifying their diets to dilute taxon‐specific defence compounds (Freeland & Janzen, 1974), if they feed in ways that suppress the local availability of preferred foods (Bryant et al., 1991; Kartzinel & Pringle, 2020; Spiller & Schoener, 1998), or if they experience competitive displacement (Pringle et al., 2019). For all of these reasons, important dietary taxa may only register in diets at low relative abundances and improving our ability to account for them is a major research priority.

Knowing that many important foods can occur at low relative abundances poses a challenge for dietary DNA metabarcoding research. These rare foods need to be documented in order to understand food webs and foraging behaviors, but common bioinformatic procedures can eliminate them from analysis. Continuing to exclude them may only serve to reinforce preconceived notions about what animals eat and inhibit new understanding of how ecology works. To fulfill the promise of dietary DNA metabarcoding by using cutting‐edge technology to characterize diets with precision requires us to overcome this challenge.

## CONFLICT OF INTEREST

Authors declare that there are no conflicts of interest.

## AUTHOR CONTRIBUTIONS

B.L.L.C. and T.R.K. conceived and designed the study; L.M.M. and C.G. provided fecal samples and microhistology data; P.T.F., V.I.S., C.V.T., and T.R.K. generated dietary DNA data; B.L.L.C. and T.R.K. led data analyses and wrote the manuscript with contributions from all authors.

## Supporting information

Appendix S1‐S2

Data S1

Data S2

Data S3

Data S4

Data S5

Data S6

## Data Availability

Illumina sequence read data and sample metadata have been made available at NCBI (BioProject accession no.: PRJNA780500; Littleford‐Colquhoun et al., 2021). Unrarefied/rarefied sequence read tables and plant taxonomy information have been made available at Dryad (https://doi.org/10.5061/dryad.kwh70rz4s). All bioinformatic scripts are available at Zenodo (10.5281/zenodo.5703310).
