# Supplementary material for: The precautionary principle and dietary DNA metabarcoding: Commonly used abundance thresholds change ecological interpretation
Source: Mol Ecol. 2022 Jan 30;31(6):1615–26. doi: 10.1111/mec.16352 (PMC9303378; doi:10.1111/mec.16352)
Supplement: Supplementary file 1 — Appendix S1‐S2 [file MEC-31-1615-s006.docx]

**Supplementary Materials for:**

*The precautionary principle and dietary DNA metabarcoding: commonly used abundance thresholds change ecological interpretation*

**Authors**: Bethan L. Littleford-Colquhoun^1,2*^, Patrick T. Freeman^1,2^, Violet I. Sackett^1,2^, Camille V. Tulloss^1,2^, Lauren M. McGarvey^3^, Chris Geremia^3^, Tyler R. Kartzinel^1,2*^

**Affiliations**:

^1^Department of Ecology and Evolutionary Biology, Brown University, Providence, RI 02912, USA

^2^Institute at Brown for Environment and Society, Brown University, Providence, RI 02912, USA

^3^Yellowstone Center for Resources, Yellowstone National Park, Mammoth Hot Springs, WY 82190

***Corresponding Authors**: [bethan_littleford-colquhoun@brown.edu](mailto:bethan_littleford-colquhoun@brown.edu); [tyler_kartzinel@brown.edu](mailto:tyler_kartzinel@brown.edu)

**Appendix S1**

*Dietary profiles of specialists and generalists*

Animal diets are generally characterized by highly skewed, concave distributions (Forister et al., 2015). This can be approximated by a power law such as the Pareto distribution, which is a continuous probability density function: *f*(*x*) = α / *x*^α+1^, where 𝑥 is the random variate and α is the shape parameter of the distribution.

To simulate an ideal diet profile, we used the Pareto distribution to generate a function of the probability that a consumer ate each of *N* food taxa present within an environment (Supplementary Figure 1). As the Pareto distribution is used to model heavy-tailed (skewed) phenomena, when this distribution is applied to *N* taxa, many taxa will have a low probability of selection while a few will have a high probability of selection. The shape parameter, α (also known as the ‘tail index’), is used to adjust the skewness of the distribution and thus the degree to which a subset of taxa has this high probability of selection (Figure S1). By altering α, and thus the shape of the Pareto distribution’s tail, the probability distribution of *N* food-taxa can be manipulated to simulate dietary profiles of specialist and generalist consumers.

In our simulations, we considered a set of three hypothetical consumers with access to an identical food base comprising 100 potentially suitable food taxa. We assumed that each consumer differed only in the specificity with which it consumed each food taxon. For each consumer, the probability that each of the 100 food taxa would be selected was calculated using the R package “Pareto” v.2.2.1 (Riegel, 2018). This function requires three parameters; *N* (number of observations; here, a constant representing the 100 food-taxa available within the environment), t (thresholds of the Pareto distribution; set to a constant value of 1), and alpha (α; the shape parameter of Pareto distribution to be varied). To generate diet profiles that differed in their degree of specialism, we defined probability distributions using different skewness values to represent a specialist (α = 0.20), intermediate (α = 0.35), and generalist (α = 1.00) diet (Figure S2). From each of these probability distributions, we made 25,000 draws of taxa to simulate the number of sequence-reads commonly obtained per sample using an Illumina MiSeq for DNA metabarcoding. By drawing a constant number of sequences in this simulation, we thus assumed that all initial data generation and processing steps—such as the removal of any low-quality sequences, chimeras, or obvious errors—were undertaken separately and result in dietary profiles with equal information content. After drawing from these three distributions, the specialist’s diet profile comprised a much narrower subset of the 100 available resources compared to the intermediate and generalist consumer.

*Dietary richness and percentage loss calculations*

The relative read abundance (RRA) of a taxon is the proportion of sequences representing that taxon in a sample divided by the final number of sequences assigned to that sample. To apply RRA thresholds, we converted sequence read counts into RRA and then applied RRA-based thresholds in 0.2% increments from 0% to 5% to remove rare taxa from each of the three dietary profiles. Higher RRA thresholds remove more food-taxa from the dataset compared to lower RRA thresholds. Dietary richness is defined as the number of food taxa within each sample after filtering, and was calculated for each sample across each threshold. In addition, we calculated the percent loss of dietary richness from the ‘true’ starting dietary richness (i.e., each threshold compared to the 0% RRA threshold)*.*

**Appendix S2**

*Dietary profiles from Yellowstone National Park*

We used illustrative dietary DNA metabarcoding data from bison (*Bison bison*) and bighorn sheep (*Ovis canadensis*) in Yellowstone National Park. The Park is a ~5,600 km^2^ expanse of protected land in northwestern Wyoming that extends into parts of Montana and Idaho, forming a core wilderness in the Greater Yellowstone Ecosystem. Within Yellowstone, vegetation is heavily influenced by elevation: low elevations are dominated by grasses and shrubs, middle elevations are dominated by Lodgepole Pine forests, and the highest subalpine elevations are populated with spruce and fir species (Marston & Anderson, 1991). There are also pronounced seasonal changes in vegetation availability: emergence begins in the spring and vegetation senescence occurs in the fall, with winter bringing a snow-covered period where vegetation is scarce (Notaro et al., 2019). Thus, the diversity of food plant taxa available to ungulates is maximized in summer and minimized in winter. These large herbivores follow distinct migratory patterns that are driven by the seasonal changes in vegetation availability (Fryxell & Sinclair, 1988). Migratory species often spend winter (December to February) in lower elevation areas, and then move to higher elevation areas in spring (March to May) and summer (June to August), following the “green wave” of sprouting plants and grasses, before returning towards wintering grounds during the fall (September to November) and winter (December to February). Bison feed primarily on grasses and other graminoids, forbs (herbaceous, broad-leafed plants), and browse (woody plants) throughout the year, while bighorn sheep feed primarily on grasses during the spring and summer, and forage on shrubby plants in the fall and winter.

*Sample collection and DNA extraction*

Sampling focused on the herds of GPS-collared adult female bighorn sheep and bison occurring over 889 km^2^ of the park. Most samples came from the northern section of the park near Lamar Valley, with one sample from the central region near Hayden Valley. Fresh fecal samples deposited by individuals within herds associated with GPS-collared animals were collected in an unused plastic zipper bag between December 2016 and October 2017 and stored at -80°C. Each collection comprised an approximately equal volume of thoroughly mixed material from 1 to 5 dung samples to provide a thorough representation of foods being consumed by the group. The analysis presented here is based on samples collected between the end of December 2016 and the end of March 2017 (winter) and between the beginning of June to the end of August 2017 (summer). These samples were transported to Brown University where DNA extraction and analysis took place. Total genomic DNA was extracted from a subsample of each collection (~0.2 mg) using the Zymo Quick-DNA Fecal/Soil Microbe Miniprep Kit (Zymo Research). Five extraction blanks were used to monitor for potential cross-contamination in the laboratory.

*Plant DNA metabarcoding*

We amplified the P6 loop of the chloroplast *trn*L (UAA) intron, which is able to identify a broad array of plant taxa (Taberlet et al., 2007). We performed PCR in 25 μL reactions using 2 mM MgCl_2_, 200 μM each dNTP, 0.2 μM each primer [trnL(UAA)g/trnL(UAA)h], Platinum Taq DNA polymerase, and 2 μL of DNA extract. The *g* and *h* primers were modified to contain an adapter overhang. Thermocycling followed a program of initial denaturation at 95°C for 5 min, followed by 35 cycles of 95°C for 30 s, 55°C for 40 s, and 72°C for 1 min, with a 10 min final extension at 72°C. We verified PCR success by running 5 μl of PCR product on a 2.0% agarose gel. Initial amplicons were then sent to the University of Rhode Island sequencing core where multiplex identification (MID) tags and P5/P7 adapters were annealed to the overhang adapter in a second-round PCR to enable Nextera Illumina sequencing. This enabled the pooling of 192 uniquely identifiable PCR products. Libraries were pooled at equimolar concentrations and sequenced on the Illumina MiSeq platform with 150 x 150 bp paired-end chemistry. We sequenced 5 positive and 5 negative controls from our PCRs. The resulting sequences were demultiplexed and adapter sequences were removed for diet analysis.

*DNA metabarcode quality control and species identification*

Fastqc (www.bioinformatics.babraham.ac.uk/projects/fastqc) was used to check the quality of all raw Illumina reads. The function *obicut* was used to trim reads with a minimum quality threshold of 30 using the program Obitools (Boyer et al., 2016) and primers were removed from forward and reverse reads using *cutadapt* (Martin, 2011). All further sequence processing was performed using Obitools. Forward and reverse sequences were aligned using the *illuminapairedend* command with a minimum alignment score of 40, and only joined sequences were retained. The 5 extraction blanks, 5 positive controls, and 5 negative controls were then removed from the dataset. A median of 659 reads per sample were generated in the extraction blanks and a median of 674 reads per sample were generated in the PCR negative controls. For the remaining 35 fecal samples (bison: *N* = 21, bighorn sheep: *N* = 14), we used the *obiuniq* command to group identical sequences and tally them within samples, enabling us to quantify the RRA of each sequence. Sequences that occurred ≤2 times overall or that were ≤8 bp were discarded. Sequences were considered to be likely PCR artifacts if they were highly similar to another sequence (1 bp difference) and had a much lower abundance (5%) in the majority of samples in which they occurred; we discarded these sequences using the *obiclean* command. All 35 samples were retained and they cumulatively represented 1,175,453 high-quality sequence reads.

To identify the remaining plant sequences, we constructed a reference plant DNA library based on *trn*L-P6 sequences available from the global European Molecular Biology Laboratories (EMBL) database (release 143). We extracted well-identified sequences from EMBL using the *ecoPCR* command. We then compared our dietary DNA sequences to this reference library using the *ecoTag* command. We assigned species identities to dietary DNA sequences based on exact matches (100% identity) to EMBL reference sequences, excluding sequences that had no counterpart in the reference library. Following all processing steps, a total of 1,071,130 of high-quality reads (91.1%) were mapped to the reference library across all 35 fecal samples. This resulted in a median of 93.2% (63.6% to 98.3%) mapped sequence reads per sample and yielded a median of 30,278 reads per sample (21,143 to 38,298 reads per sample). Across all 35 animal samples, 357 plant sequences were identified with a median of 65 reads per sequence (1 to 97,964 reads per sequence).

*Comparative analysis of RRA thresholds*

Our sequencing and bioinformatics analysis generated a file representing a table of sequence counts per food-taxon per sample (available at Dryad DOI:10.5061/dryad.kwh70rz4s), a table containing the taxonomy of each food-taxon (available at Dryad DOI: 10.5061/dryad.kwh70rz4s), and a table containing the sample metadata (available at NCBI BioProject accession number: PRJNA780500). We used these files to create a phyloseq object for analysis using the *phyloseq* package in R (McMurdie & Holmes, 2014). All samples were then rarefied to an even depth of 21,143 sequences per sample. Sequence read counts were converted into RRAs per sample by dividing each cell in the read count table by this total number of sequences per sample (*N* = 21,143).

To assess how RRA thresholds influence the rank order of individual dietary richness, we used four representative samples collected in summer (2 bighorn sheep, 2 bison). We then removed taxa from each sample that did not exceed each RRA threshold, which we increased in increments of 0.2% from 0% to 5%. Dietary richness was calculated per sample per RRA threshold using the *vegan* package in R (Oksanen et al., 2007). For each sample, we plotted: the dietary profile prior to removal of any low-abundance taxa (Figure 2a, 2d), the dietary richness of each sample at each RRA threshold (Figure 2e), and the percent loss of apparent dietary richness with each RRA threshold (Figure 2f).

To assess how RRA thresholds influence well-studied differences in dietary richness between bighorn sheep and bison in winter and summer, we calculated mean dietary richness across samples. Sample sizes per species per season ranged from 4 to 11 (bison: winter*_N_* = 11, summer*_N_* = 10; bighorn sheep: winter*_N_* = 4, summer*_N_* = 10). For the sake of comparison, we calculated mean dietary richness using each of four commonly used RRA thresholds in the literature: 0%, 0.1%, 1% and 5%.

As above, to determine how RRA thresholds influence the apparent total dietary niche breadth of each population, we calculated total-population dietary richness for bighorn sheep and bison in winter and summer months based on the same RRA thresholds (0%, 0.1%, 1%, and 5%). In order to account for differences in sample sizes between seasons and species, we performed sample-based rarefaction and extrapolation using the iNEXT package v2.0.20 (Hsieh et al., 2016). We based our comparisons on the equivalent of double the minimum seasonal sample size for each species (8 for bighorn sheep and 20 for bison). The iNEXT function was run using food-taxa incidence frequencies with 10 knots, 10,000 bootstraps, and 95% confidence intervals.

*Calculation of Hill numbers*

Hill numbers were applied to both simulated and real dietary data. For the simulated data (Figure 4a), four representative samples from the Yellowstone data (Figure 4b), and the average population-level average of bighorn sheep and bison from Yellowstone (Figure 4c), Hill numbers were calculated using the *vegan* package in R. As rare taxa can be upweighted or downweighted using the scaling parameter (*q*), we calculated *q* values in increments of 0.2 from 0 to 2 (Figures 4 a-c).

To calculate Hill numbers for total population-level estimates for bighorn sheep and bison, we used the point estimation function (estimateD) in the iNEXT package to compute diversity estimates with *q* = 0, 1, 2 for a particular level of sample size (base="size"). We based our comparisons on the equivalent of double the minimum seasonal sample size for each species (8 for bighorn sheep and 20 for bison) and 95% confidence intervals (Figure 4d).

*Comparison of metabarcoding and microhistological data*

We compared DNA metabarcoding and microhistology data. Fecal samples collected during January to March (winter months) and June to August (summer months) in 2017 were sent to Washington State University (Pullman, WA, USA) to determine the botanical composition of samples using microhistology. Multiple fecal samples collected from the same herd within the same season were composited into a single sample per season. Two microscopic slides were made from each composite fecal sample and 25 randomly placed microscopic views were made from each slide, for a total of 50 views per composite sample. Forage classes were identified as grass, sedge/rush, lichen/moss, forbs, shrubs, conifers, lichen and moss. Diets were compiled as percent cover of plant fragments by genus of only the top 6 to 12 genera within each sample and further identified to species when possible. Less common genera, as determined by microhistologists at Washington State University, were excluded from analysis; this is a common strategy employed during microhistological analyses. Microhistology allows taxa to be identified and quantified but requires accurate identification of partially digested plant fragments and thus tends to overemphasize less-digestible taxa.

**References**

Boyer, F., Mercier, C., Bonin, A., Le Bras, Y., Taberlet, P., & Coissac, E. (2016). obitools: A unix‐inspired software package for DNA metabarcoding. *Molecular ecology resources*, *16*(1), 176-182.

Forister, M. L., Novotny, V., Panorska, A. K., Baje, L., Basset, Y., Butterill, P. T., . . . Diniz, I. R. (2015). The global distribution of diet breadth in insect herbivores. *Proceedings of the National Academy of Sciences*, *112*(2), 442-447.

Fryxell, J., & Sinclair, A. (1988). Causes and consequences of migration by large herbivores. *Trends in Ecology & Evolution*, *3*(9), 237-241.

Hsieh, T., Ma, K., & Chao, A. (2016). iNEXT: an R package for rarefaction and extrapolation of species diversity (H ill numbers). *Methods in Ecology and Evolution*, *7*(12), 1451-1456.

Marston, R. A., & Anderson, J. E. (1991). Watersheds and vegetation of the Greater Yellowstone Ecosystem. *Conservation Biology*, *5*(3), 338-346.

Martin, M. (2011). Cutadapt removes adapter sequences from high-throughput sequencing reads. *EMBnet. journal*, *17*(1), 10-12.

McMurdie, P. J., & Holmes, S. (2014). Waste not, want not: why rarefying microbiome data is inadmissible. *PLoS Comput Biol*, *10*(4), e1003531.

Notaro, M., Emmett, K., & O’Leary, D. (2019). Spatio-temporal variability in remotely sensed vegetation greenness across Yellowstone National Park. *Remote Sensing*, *11*(7), 798.

Oksanen, J., Kindt, R., Legendre, P., O’Hara, B., Stevens, M. H. H., Oksanen, M. J., & Suggests, M. (2007). The vegan package. *Community ecology package*, *10*(631-637), 719.

Riegel, U. (2018). Matching tower information with piecewise Pareto. *European Actuarial Journal*, *8*(2), 437-460.

Taberlet, P., Coissac, E., Pompanon, F., Gielly, L., Miquel, C., Valentini, A., . . . Willerslev, E. (2007). Power and limitations of the chloroplast trn L (UAA) intron for plant DNA barcoding. *Nucleic Acids Research*, *35*(3), e14-e14.

**SUPPLEMENTARY FIGURES**


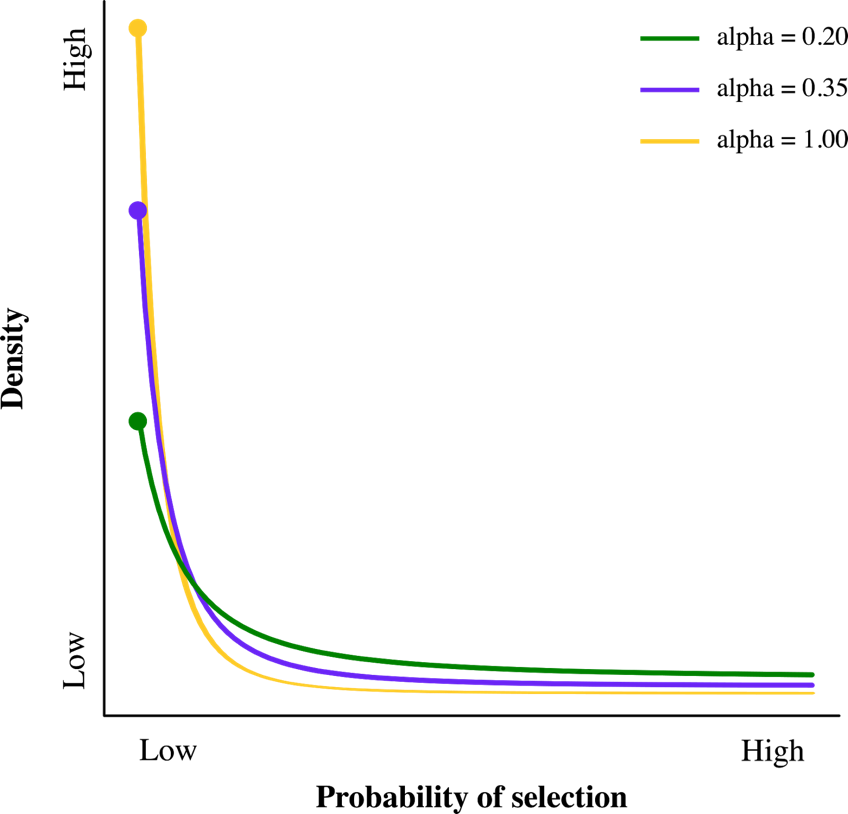


**Figure S1.** Illustration of how the probability density function of the Pareto distribution varies with the shape parameter, alpha (α). When α = 1.00, there is a high density of low probability values and a low density of high probability values. Conversely, when α = 0.20, there is a lower density of low probability values and a higher density of high probability values. The shape parameter changes the probability of selection (Figure S2).


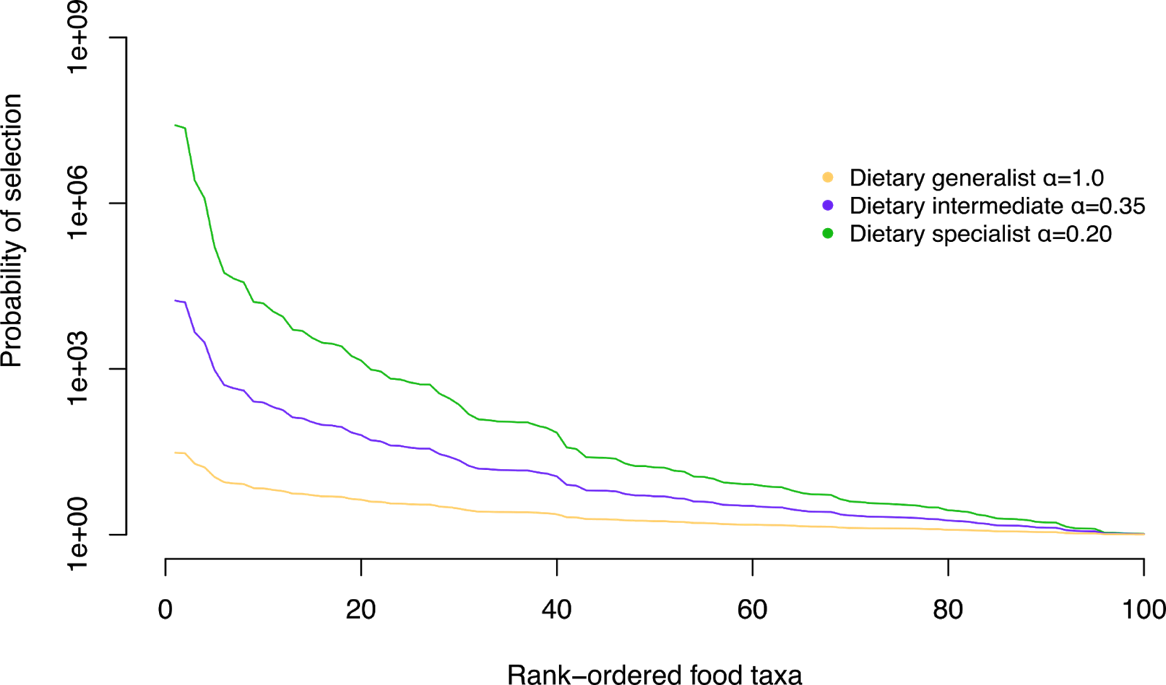


**Figure S2.** Line plot showing the rank-ordered probability of selection of 100 food taxa for the three consumers in our simulation study: a generalist, intermediate, and specialist feeder. All three consumers had access to an identical food base and only differed in the specificity with which they consumed each food taxon as defined by the shape parameter (α) of the Pareto distribution. To generate dietary profiles that differed in degree of specialism, we used different skewness values to represent a specialist (α = 0.20), intermediate (α = 0.35), and generalist (α = 1.00) using the R package “Pareto” v.2.2.1 (Riegel 2021).

 
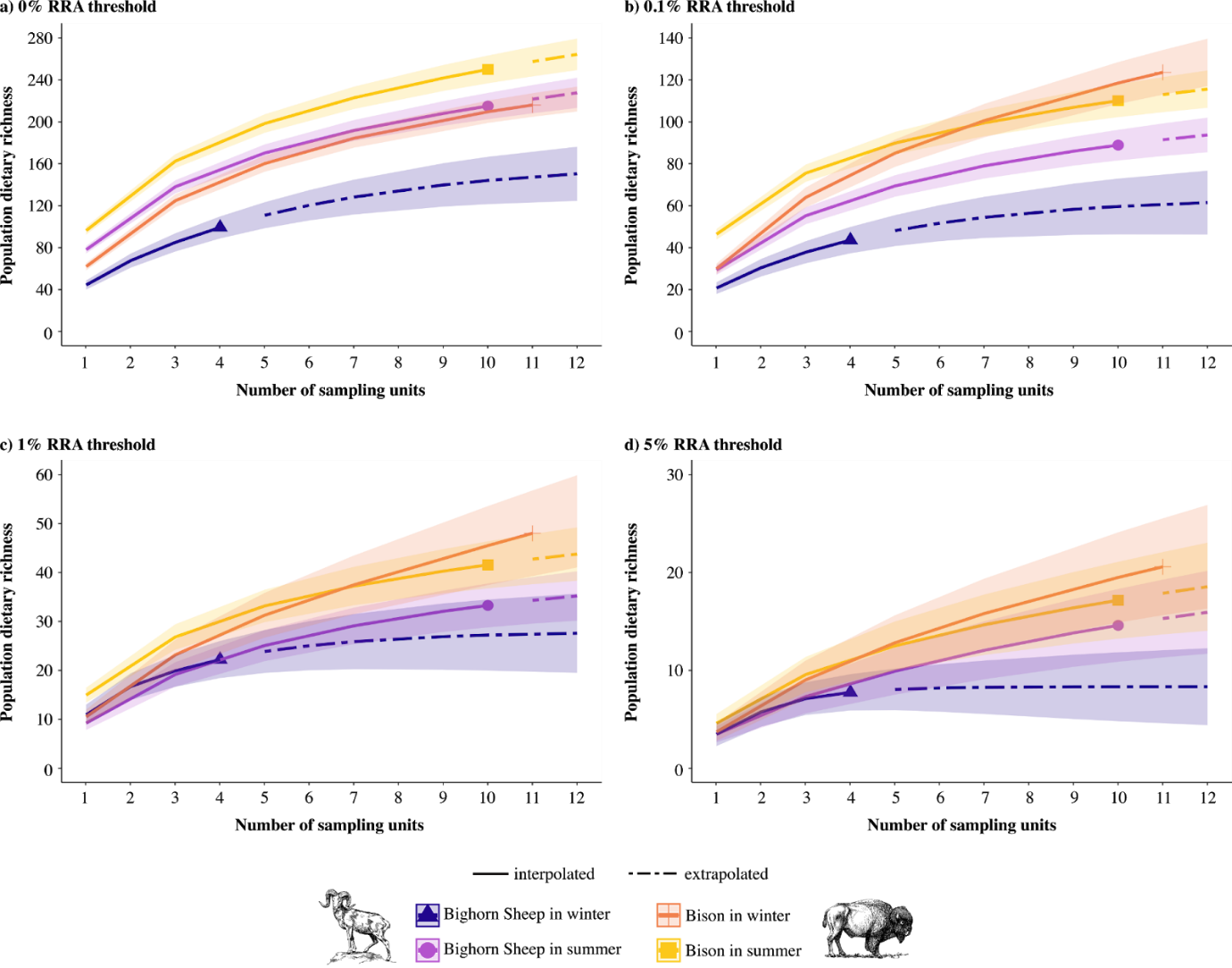
 **Figure S3.** Using RRA thresholds to filter low-abundance sequences altered apparent population-level dietary richness for Yellowstone bighorn sheep and bison. Sample size-based rarefaction (solid lines) and extrapolation (dashed lines) curves show estimates of total population-level dietary richness in winter (darker colors) and summer (lighter colors) for bighorn sheep and bison using **(a)** 0%, **(b)** 0.1%, **(c)** 1%, and **(d)** 5% RRA threshold. In all plots, the 95% confidence intervals (shaded areas) were calculated based on 10,000 bootstrap replicates.

**
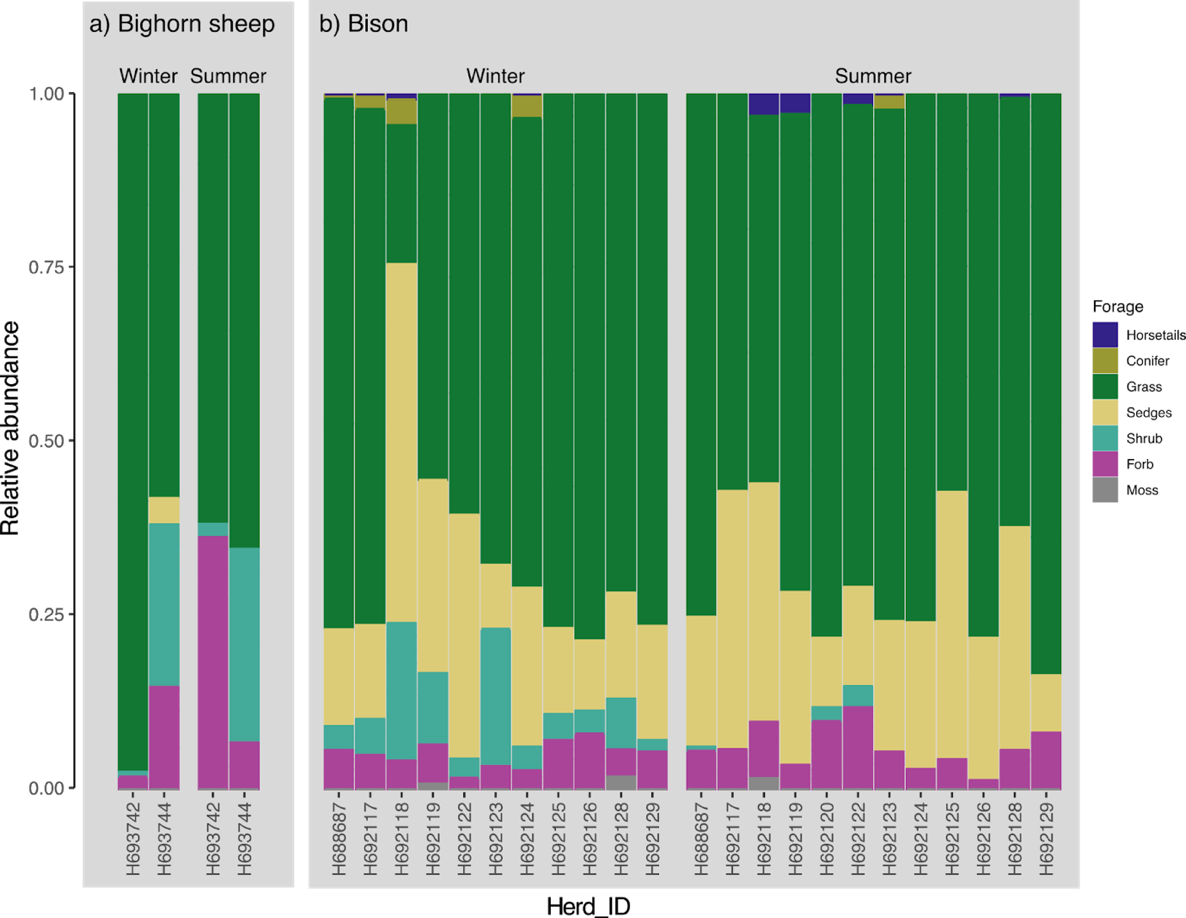
**

**Figure S4.** Microhistology data showing the proportion of horsetails, conifers, grasses, sedges/rushes, shrubs, forbs, and mosses identified from the same herds of (a) bighorn sheep and (b) bison that we studied using DNA metabarcoding in winter and summer. These microhistology samples were pooled across animals and collection dates to represent a composite seasonal diet profile for each herd, and thus we obtained fewer representative diet profiles using microhistology compared to DNA metabarcoding.
